# A multifaceted primary care practice-based intervention to reduce ED visits and hospitalization for complex medical patients: A mixed methods study

**DOI:** 10.1371/journal.pone.0209241

**Published:** 2019-01-02

**Authors:** Tara O’Brien, Noah Ivers, Onil Bhattacharyya, Andrew Calzavara, Laura Pus, Geetha Mukerji, Steven M. Friedman, Howard Abrams, Ian Stanaitis, Gillian A. Hawker, Pauline Pariser

**Affiliations:** 1 Department of Medicine, University of Toronto, Toronto, Ontario, Canada; 2 Women’s College Hospital, Toronto, Ontario, Canada; 3 Department of Family and Community Medicine, University of Toronto, Toronto, Ontario, Canada; 4 Institute for Clinical Evaluative Sciences, Toronto, Ontario, Canada; 5 University Health Network, Toronto, Ontario, Canada; University of Mississippi, UNITED STATES

## Abstract

**Background:**

The management of complex, multi-morbid patients is challenging for solo primary care providers (PCPs) with limited access to resources. The primary objective of the intervention was to reduce the overall rate of Emergency Department (ED) visits among patients in participating practices.

**Methods and findings:**

An interrupted time series design and qualitative interviews were used to evaluate a multifaceted intervention, SCOPE (Seamless Care Optimizing the Patient Experience), offered to solo PCPs whose patients were frequent users of the ED. The intervention featured a navigation hub (nurse, homecare coordinator) to link PCPs with hospital and community resources, a general internist on-call to provide phone advice or urgent assessments, and access to patient results on-line. Continuous quality improvement (QI) strategies were employed to optimize each component of the intervention. The primary outcome was the relative pre-post intervention change in ED visit rate for patients of participating practices compared with that for a propensity-matched control group of physicians over the contemporaneous period. Themes were identified from semi-structured interviews on PCP’s experiences and influential factors in their engagement. Twenty-nine physicians agreed to participate and were provided access to the intervention over an 18-month time period. There were a total of 1,525 intervention contacts over the 18-months (average: 50.6±60.8 per PCP). Both intervention and control groups experienced a trend towards lower rates of ED use by their patients over the study time period. The pre-post difference in trend for the intervention group compared to the controls was not significant at 1.4% per year (RR = 1.014; p = 0.59). Several themes were identified from qualitative interviews including: PCPs felt better supported in the care of their patients; they experienced a greater sense of community, and; they were better able to provide shared primary-specialty care.

**Conclusions:**

This multifaceted intervention to support solo PCPs in the management of their complex patients did not result in a reduced rate of ED visits compared to controls, likely related to variable uptake among PCPs. It did however result in more comprehensive and coordinated care for their patients. Future directions will focus on increasing uptake by improving ease of use, increasing the range of services offered and expanding to a larger number of PCPs.

## Introduction

An aging population and medical progress have led to increasing numbers of people living with one or more chronic conditions [1–5], who are at increased risk for emergency department (ED) visits, and hospital admissions [6–8]. As a result, primary care providers (PCPs) are facing increasingly complex case-loads and experiencing greater challenges meeting the health care needs of their patients [9,10].

More comprehensive team-based primary care has been shown to impact healthcare utilization [11–13]. For example, The Nuka System of Care model in Southcentral Alaska, created comprehensive team-based primary care to support the local community, with specialist consultation brought into the team as needed [14]. This model has been associated with improved health outcomes and a decrease in healthcare utilization [15]. Similarly, the CareMore Model, which provides primary care by interdisciplinary teams with support from internists during hospitalizations and periods of health deterioration, achieved lower hospitalization rates and inpatient length of stay compared to the Medicare average [16]. These models support enhancing primary care through interdisciplinary teams with access to more acute care support. Although both of these comprehensive primary care models appear to improve health outcomes while decreasing healthcare utilization, neither have been rigorously evaluated.

In this quality improvement (QI) initiative we aimed to increase PCP capacity and system integration with SCOPE (Seamless Care Optimizing the Patient Experience), an intervention which supports solo PCPs with a ‘virtual’ team of health professionals that provide direct clinical advice and enhance care coordination to prevent clinical crises that result in ED visits and hospitalizations. The primary objective was to reduce the overall rate of ED visits among patients in participating practices.

## Materials and methods

We developed an intervention informed by evidence and tailored to address local barriers [17] and evaluated the intervention using an interrupted time series with propensity matched control group.

### Intervention development

Semi-structured one-on-one interviews were conducted with eleven solo PCPs whose patients had collectively experienced ≥100 ED visits to the local hospitals over a one‐year period to garner their opinions regarding resources that would assist them in caring for their complex medical patients. This was supplemented by root cause analyses performed in a convenience sample of medical inpatients (n = 3) admitted for an ambulatory care sensitive condition (ACSC) [18], where appropriate ambulatory care may prevent hospitalizations e.g. congestive heart failure, COPD, atrial fibrillation, and family members (n = 1) to ascertain reasons for ED use. Through interviews, participants were asked to describe their reasons for hospitalization, including details regarding the events leading up to the admission, such as whether or not they had seen their family doctor, and to provide their opinion about whether or not the hospital admission might have been prevented, and if so, how. Finally, a systematic review of the literature was performed to ascertain effective QI interventions for the coordination of care of frequent users of the health care system [19]. The results identified the following services as having potential impact on ED use in patients with chronic disease: comprehensive and accessible information on services available, greater availability of homecare, access to hospital records, and timely access to urgent diagnostic and specialty assessment. These results informed the design of a multi-faceted intervention, whereby participating PCPs were offered a single point of contact to access a Navigation Hub and/or a general internal medicine (GIM) consultant. Plan-Do-Study-Act Cycles [20] were conducted to optimize each component of the multi-faceted intervention in an iterative manner based on process metrics and ED visits, as well as feedback from participating PCPs and the SCOPE operations team.

### SCOPE intervention

The Navigation Hub comprised of a hospital-based nurse and a care coordinator from a community service agency who fielded questions (by telephone and email) from participating PCPs and/or their clerical support staff from 9AM to 5PM on weekdays. It aimed to facilitate timely access to specialty medical care, diagnostic testing, and community and hospital services, including intensive case management for patients with complex care needs. When PCPs contacted the Navigation Hub, they were informed services they may not have been aware of which may be more appropriate for their patients, and might be advised of services or specialists with shorter wait times. As a result, patients in the intervention group for whom PCPs called the Navigation Hub may have received appropriate services slightly faster than the control PCPs.

A GIM consultant was available by phone to answer clinical queries as well as provide urgent patient assessments in a short stay medical unit (Acute Ambulatory Care Unit, AACU). The AACU is staffed by a GIM physician, nurses, as well as a pharmacist and can provide assessment and work up of acute medical illness or management of exacerbations of chronic medical conditions. In the AACU, there is access to urgent blood work, rapid diagnostic imaging, non-invasive cardiac testing, subspecialty support, as well as the ability to give IV therapies including transfusions. To access the AACU, patients must be referred by a physician, be medically stable and able to return for outpatient follow-up. The unit does not take walk-in patients and does not have access to surgical support services. While the AACU operates 24 hours a day on weekdays, the GIM consultant was available from 8AM to 6PM. Access to a GIM consultant aimed to provide a more convenient and comprehensive approach to caring for patients who may have otherwise been referred to the ED.

In addition, PCPs received access in their offices to Patient Results Online (PRO). PRO was a web-based application providing secure access to health information from participating hospitals and lab information systems across the Greater Toronto Area, enabling timely online access to hospital-based patient records (laboratory, imaging, and consultation notes). SCOPE PCPs received access to PRO before it became widely available to physicians across the province of Ontario; the current adaptation of PRO is named ConnectingOntario.

### Eligibility

Solo PCPs whose patients’ had high rates of use of the local hospital ED overall and for ACSC were eligible for the intervention. Solo PCPs were defined as community-based PCPs without practice-based access to other health professionals. Physicians were identified using the National Ambulatory Care Reporting System (NACRS) database and high ED use was defined as those with ≥30 patients per month with ED visits for any reason and ≥10 patients per month with ED visits for ACSC. PCPs who worked exclusively in walk‐in clinics or did not practice as general or family physicians, e.g. sports medicine, palliative care practitioners, were excluded.

### Recruitment

Eligible PCPs were mailed an information letter by the partnering institutions inviting participation in a QI project focused on enhancing the quality of communication and services provided to PCPs by a local hospital and community service agency. Members of the clinical team and the study coordinator visited those who agreed to participate in their offices at a convenient time to provide a more detailed overview of the project. Interested PCPs were invited to participate in group engagement events to garner enthusiasm for participation and to build a sense of camaraderie with the SCOPE team and fellow PCP colleagues. Separate engagement events were held with the physicians’ front-line staff. Following the initial physician engagement event, written consent was obtained from those PCPs interested.

### Propensity matched controls

We used propensity score matching to obtain a comparable group of PCPs [21]. A pool of 1,090 control solo PCPs practicing in Toronto since at least 2008, whose patients exceeded 200 annual ED visits, were identified. The control group were selected from an administrative database from areas with access to the usual referral mechanism to subspecialists and acute care hospitals as well as community resources, but no access to a short stay medical unit. A propensity score was calculated based on physician characteristics: age, sex, time since graduation, specialty, primary care funding type (Fee-for-service; Family Health Group (fee-for-service plus incentives and bonuses for services to enrolled patients); Family Health Organization (blended capitation); or Comprehensive Care Model (fee-for-service plus incentives and bonuses for services to enrolled patients)), hours worked in 2012 (based on total physician claims), history of working in an ED in 2012 (yes/no), and, as measured in the pre-intervention period, annual roster size and ED visit rate. A 1:m (3≤m≤5) propensity match within 0.2 standard deviation units was used.

### Outcomes

The primary outcome was the relative change in ED visit rate for patients of participating practices–the number of ED visits by patients of a given physician divided by the total number of patients rostered to that physician compared with that for a propensity-matched group of physicians for each three month interval. As we did not expect the intervention to impact ED visits for mental health reasons or trauma, these visits were excluded from the primary outcome, but mental health visits were incorporated in our outcome in a sensitivity analysis. Secondary outcomes were: comparative hospital admission rates overall and for ACSCs and cumulative length of stay of inpatient admissions in each quarter per patient-year rostered (excluding admissions for trauma and mental health). Outcomes were examined with and without a wash-out period of three months following the start of the intervention.

Using previously validated methods developed at the Institute for Clinical Evaluative Sciences (ICES) [22, 23], the panel of adult patients (18-years or older) cared for by a given SCOPE PCP, was defined as those for whom the SCOPE physician was the physician with the highest total primary care billing claims for that patient over the prior two fiscal years. Rosters were recalculated quarterly to account for patients joining or leaving a given practice. In-patient hospitalizations were identified using the Canadian Institute for Health Information (CIHI) hospital discharge abstract database (DAD), while ED visits were obtained from the NACRS database for the period beginning three years prior to the SCOPE intervention. Billings were obtained from the Ontario Health Insurance Plan (OHIP) database of physician services. These datasets were linked using unique encoded identifiers and analyzed at ICES.

### Process measures

All contacts made to the Navigation Hub and GIM consultant were tracked, including the date, reason and outcome of the encounter. For each PCP, the pattern of contacts was examined weekly. Monthly rates of ED visits and hospital admissions were also monitored. Based on overall number of SCOPE contacts for each physician, those in the lowest quartile for contacts (low use of SCOPE), but in the highest quartile for patient ED visits (potential high need for SCOPE), were targeted for additional engagement by the SCOPE operations team [24].

### Qualitative

Semi-structured interviews were conducted with PCPs to examine their experience with SCOPE and influential factors in their engagement [25]. The research team developed the interview guide (S1 Appendix), pilot tested with team members that practice family medicine, and periodically refined based on feedback and suggested revisions from the team and emerging themes from the interviews. All interviews were audio-recorded, transcribed verbatim and coded for key themes using a grounded theory informed analytic approach [26, 27] and NVivo 10 software (QSR International, Melbourne, AU). Data regarding PCPs’ engagement with the study and perceived facilitators and challenges to participation were coded employing the constant comparative method [28, 29] and themes developed. One member of the research team conducted all interviews; other team members reviewed the coding and the themes on an iterative basis. Double coding was employed, where members of the research team independently coded a randomly selected sample of the transcripts, to validate the coding strategy and ensure consistency and comprehensiveness in data interpretation.

### Analysis

Using an interrupted time series design [30–33], the best-fit pre-intervention and post-intervention lines were determined with segmented linear regression. We calculated the change in level of outcome at the first time-point post-intervention (6-months post-intervention) and the change in slopes of the regression lines (calculated as post-intervention slope minus pre-intervention slope). The comparison between intervention and control was modeled using a generalized linear mixed model with Poisson distribution and log link. The model accounted for fixed effects of: time, whether an observation was pre- or post-intervention (“pre/post”), and whether a physician was in the intervention or control group (“case/control”), as well as all two-way interactions and a three-way interaction of pre/post by case/control by time. The three-way interaction term addressed whether the ED visit rate changed with respect to time in the intervention cohort as compared to the control group (“difference in differences”). An offset was used to account for roster size. To account for correlation of repeated measures within physicians as well as correlation arising from the case-control match, two random intercepts with unstructured covariance were included in the model. Similarly, the log of cumulative length of stay per patient-year was fit using a generalized estimating equation mixed model with normal distribution, identity link, a random intercept to account for the matched data, and unstructured correlation to account for repeated measures within physicians. A two-sided p-value of 0.05 was used for statistical significance. SAS Version 9.4 (SAS Institute Inc., Cary, NC, USA) software was used for this analysis.

### Ethical considerations

The study was approved by institutional research ethics boards at Women’s College Hospital (REB # 2012–0059) and University Health Network (REB # 12–0423), Toronto, Canada. All PCPs involved in the study provided written informed consent, including consent to participate in the intervention, engage in qualitative interviews, and linking their patient rosters at ICES.

## Results

### Participants

Of the 50 PCPs identified as having practices with high ED use, 29 consented to participate. As described in Table 1, the majority of participating PCPs were male and over the age of 50. We collected process metrics for all 29 of the consenting PCPs, but were only able to collect ED visits for 27 as two of the physicians were new to practice and did not have baseline ED visit data. We matched 126 controls to 26 SCOPE physicians; one physician was excluded as there were zero matches in the control group. After matching, standardized differences in physician characteristics (weighted to account for 1:m matching) were all less than 0.10, with the exception of age, time since graduation, and primary care funding type which had values of 0.15, 0.13, and 0.12 respectively.

**Table 1:** Primary care physician characteristics and practice profile.

Office Profile Characteristic		N = 29
Sex–n (%)	Male	24 (82.8)
	Female	5 (17.2)
Age–n (%)	30–39 years	2 (6.9)
	40–49 years	7 (24.1)
	50–59 years	8 (27.6)
	60+ years	12 (41.4)
Years in Family Practice–n (%)	≤ 10 years	2 (6.9)
	11–15 years	2 (6.9)
	> 15 years	25 (86.2)
Practice size–n (%)	≤ 2000	11 (37.9)
	2001–3000	6 (20.7)
	> 3000	12 (41.4)
Hours a week spent in patient care–n (%)	30 or fewer	5 (17.2)
	31–40	15 (51.7)
	More than 40	9 (31.1)
Practice appointment scheduling–n (%)	Same day appointment	24 (82.8)
	Planned appointment (scheduling)	23 (79.3)
	Same day walk-in (no appointment)	17 (58.6)
Uses email to connect with patients–n (%)		12 (41.4)
Certification in The College of Family Physicians (CCFP)–n (%)		17 (58.6)
Experience in the ED–n (%)		17 (58.6)
Offer after-hours coverage–n (%)		26 (89.7)
Uses Electronic Medical Record (EMR)–n (%)		21 (72.4)
Number of patients seen in ½ day–mean (range)		23.4 (12–50)
Number of hours worked per week–mean (range)		37.4 (24–56)

### Process metrics

**Fig 1:**
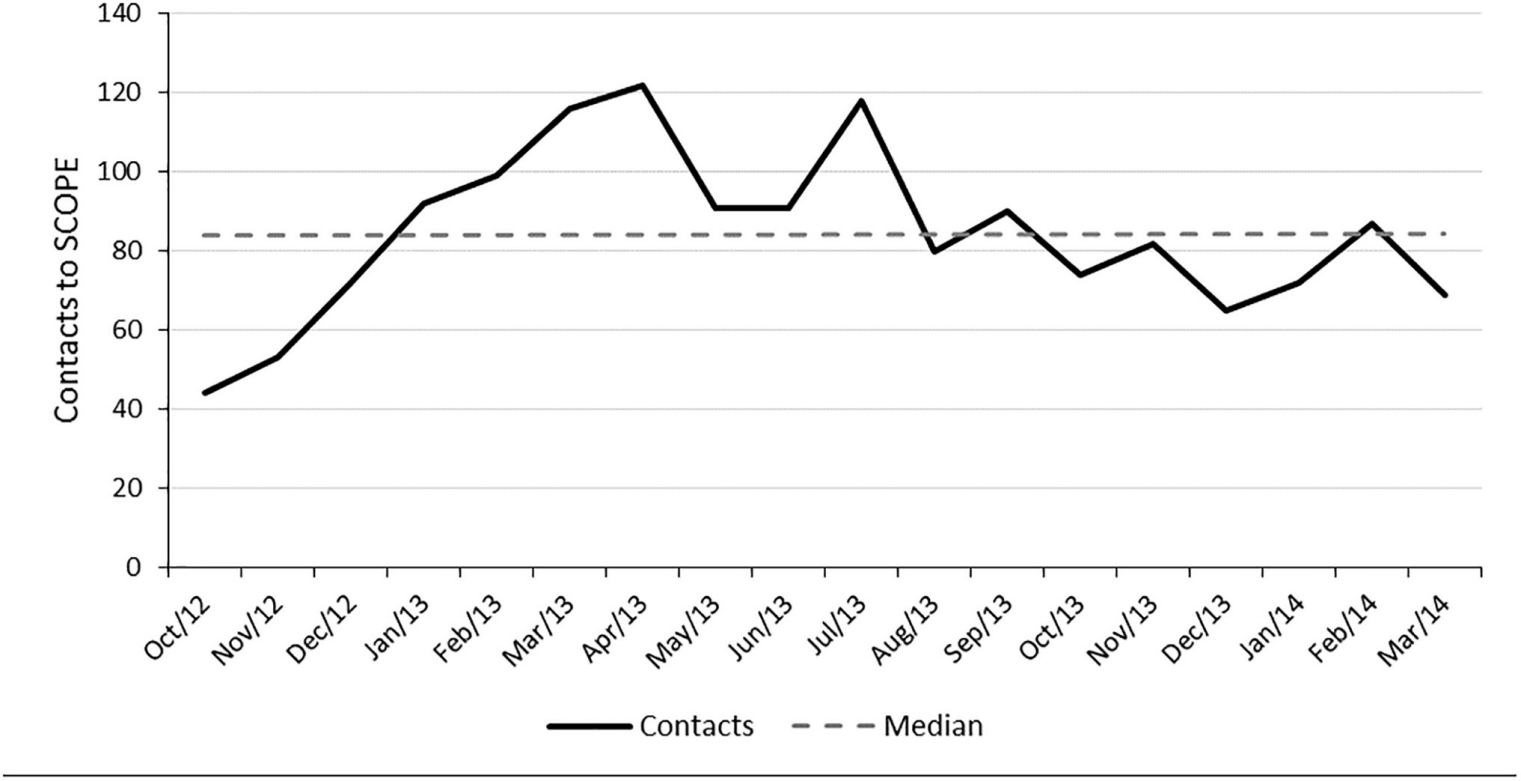
Total monthly calls to the SCOPE intervention.

The 1,525 total SCOPE contacts were plotted over time for the duration of the intervention (Fig 1). There was large variability in SCOPE usage across the PCPs. The number of contacts to SCOPE by each PCP ranged from 4 to 288 (median 31.0; IQR 21.5–51.8) over the duration of the intervention. Three PCPs accounted for 640 (42%) contacts to SCOPE, 7 PCPs made between 40–99 contacts, while 6 PCPs each made fewer than 20 contacts. The majority of the calls were made to the Navigation Nurse (n = 694, 46%) followed by the GIM consultant (n = 488, 32%) and homecare (n = 343, 23%) (S1 Fig). The reasons for contacting SCOPE varied, most common being requests for community and homecare assessments (S1 Table). Twenty-four PCPs used PRO to view 11,031 patient results (S2 Fig). PRO use varied widely (range 2–3345; median 133.0; IQR 65.8–616.3).

The most common outcomes from a call to SCOPE were referral to the AACU (n = 345, 22%) and provision of homecare services (n = 304, 19%) (S3 Fig). The most common referrals to the AACU were for chest pain and arrhythmia, cellulitis, and deep venous thrombosis. Contacts with SCOPE resulted in 282 referrals to specialists; psychiatry, addictions medicine and neurology were most common (S4 Fig).

Use of SCOPE varied significantly across providers, we identified the subset of PCPs who did not use SCOPE frequently and had high ED rates (PCP call volume compared with total patient ED visits at a local hospital on a monthly basis), and we approached them to understand how they might further be engaged and if they experienced any barriers in their use of SCOPE. Feedback led to the introduction of a number of new strategies to increase engagement including targeted methods (individual office visits and conversations), non-direct approaches (automated fax referral system, monthly ED reports, more frequent PCP events and communications featuring available services), and addition of new services (resources for ethnic groups, addiction/substance use clinic). By targeting these PCP’s who appeared to be disproportionately under using the service, and whose patients had greater number of ED visits, we were able to identify operational efficiencies and areas for improved PCP engagement.

### Comparison to propensity matched cohort

**Fig 2:**
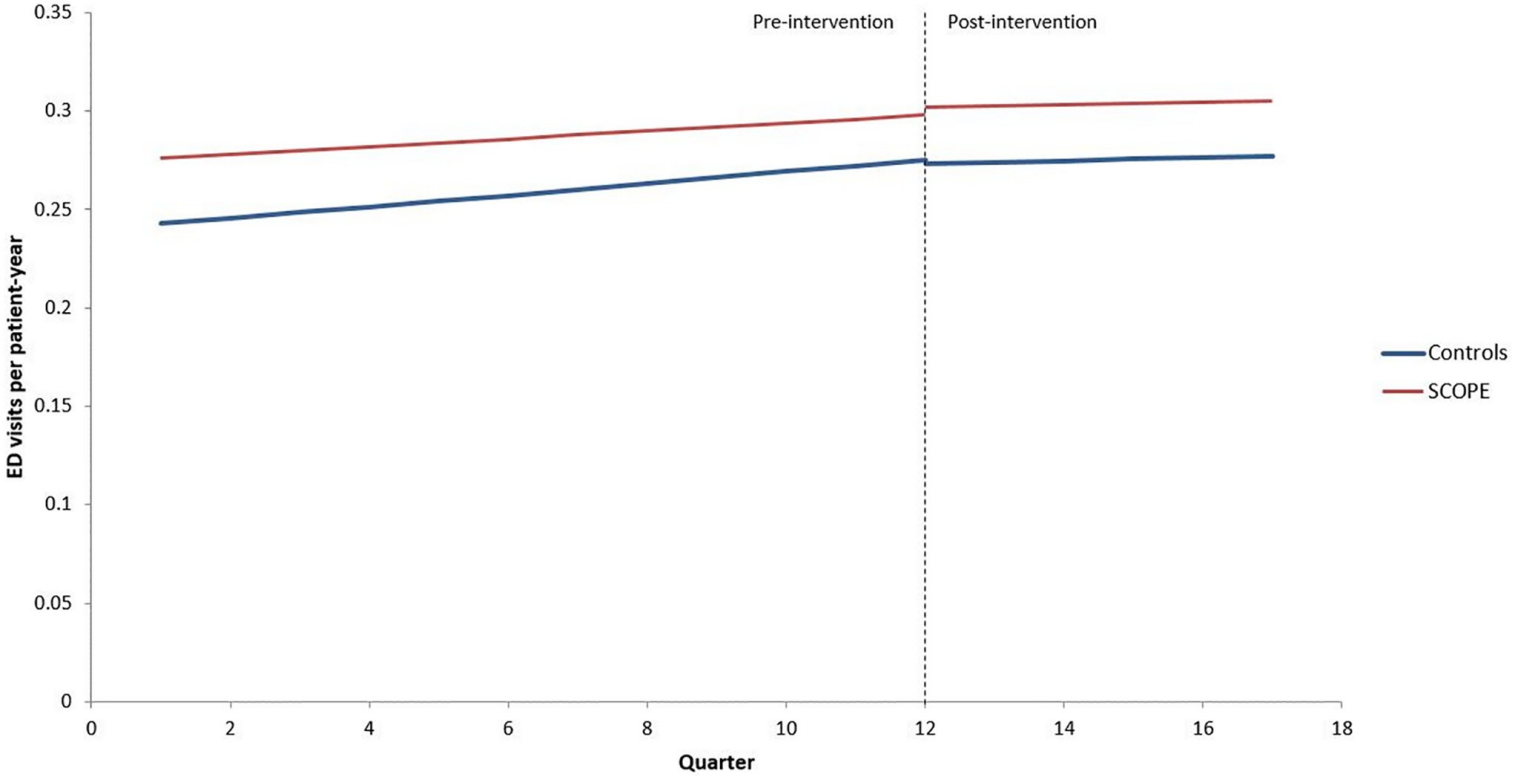
ED visit rates quarterly from 3 years pre- to 18 months post-intervention (Oct 1, 2009 –Mar 31, 2014).

The primary outcome over time for both SCOPE and control PCPs is illustrated in Fig 2. In the three years prior to intervention, the ED visit rate increased more slowly quarter-over-quarter for the SCOPE group (Relative rate (RR) 1.028 per year; p<0.0001) than for the control group (RR 1.049; p<0.0001). For the period from three months to 18-months post-intervention, the SCOPE group ED visit rate increased at 0.8% per year (RR = 1.008; p = 0.71) while the trend for control group was similar (RR = 1.012; p = 0.24). The pre-post difference in trend for the SCOPE group as compared to the controls was not significant at 1.4% per year (RR = 1.014; p = 0.59). The difference in the ED visit rate at the start of intervention versus three months post-intervention was not significant for the SCOPE (RR = 1.01; p = 0.53) or control group (RR = 0.99; p = 0.38). There was no significant difference in ED visit trends between SCOPE physicians and controls when comparing the post-intervention period to the pre-intervention period (“pre-post”). Analyses incorporating mental health visits and examining in-patient length of stay similarly showed no significant pre-post change in trend for SCOPE physicians relative to the control physicians. There was also no significant pre-post change in trend for admissions. Including the first three months of post-intervention data without a wash-out period in the model yielded similar results.

### Qualitative results

Twenty-two of the 29 PCPs were interviewed (76%). Key themes identified from the interviews suggest that PCPs experienced an increased sense of community with the SCOPE team. PCPs felt better supported in the care of their patients and were better able to provide shared primary-specialty care.

“I think it was a very good idea that we are not left outside as a GP, that we are incorporated with the hospital, that we have more connection and more cooperation with the specialists, and to have access to my patients’ results. That was the main reason. We have to cooperate. These days you cannot just be isolated family physician practicing without being in contact with the other health professionals.” (KI005).

Importantly, PCPs felt they were able to maintain their professional identity while involving others in the care of their patients, that SCOPE helped PCPs to recognize that they did not need to bear the entire burden of responsibility for their complex medical patients’ care in such an independent manner.

“I think [SCOPE] has made me realize that we [were] trying to do too much before and there are ways to share the load to make things easier for the patients and the doctor.” (KI010).

## Discussion

This study examined the impact of a unique multifaceted intervention targeted at solo PCPs on ED visits over an 18-month time period. Using a time series analysis with a propensity matched physician cohort, a similar, significant decrease in the rate of ED visits pre-post intervention was seen in both the intervention physician practices and controls. Of note, had we not done the propensity matched cohort, we would have erroneously concluded that our intervention reduced the rate of ED visits. This supports the need for rigorous innovative evaluation methods for QI studies.

A concurrent qualitative study suggests SCOPE increased primary care capacity by linking solo PCPs in the community to a virtual interdisciplinary team including case management and home based care [25]. In addition, SCOPE created new linkages between acute and community institutions to facilitate better care pathways and improved access for patients. The result was a flexible platform for linking services based on the needs of a given community. Unlike other team based primary care models, SCOPE provided immediate access to subspecialty advice and support. Access to PRO improved information sharing between hospital and community providers. PCPs who used SCOPE felt that their patients received more comprehensive and coordinated care with more appropriate use of healthcare resources.

The literature on interventions to reduce ED use shows variable impact [34–36], and studies often target the ED or the patient, but relatively few target the PCP exclusively. The lack of impact on PCP’s ED visit rate in this study was likely multifactorial, including low number of PCP contacts on average, as well as patient’s bypassing their PCP and directly visiting the ED with no opportunity for the PCP to access the service. In this study, 3 PCPs accounted for 42% (n = 640) contacts and the remaining 26 PCPs on average made approximately 34 contacts each over 18 months. SCOPE staff estimate that one third of contacts results in an ED visit being avoided, thus we would anticipate that for the majority of PCPs in the study, SCOPE only prevented approximately 11 ED visits per physician over an 18 month period with an average rate of 737 ED visits per physician during that time. This is clearly too small a number to impact the overall ED visit rate per practice compared to controls. Finally, this study did not explore the practice patterns of PCP office staff who are important gatekeepers with regards to answering the phone and providing appointments [37]. Patients present to the ED for non-urgent problems for a range of reasons, including accessibility of the PCP [38–41], SCOPE did not address the health system or patient-level factors contributing to ED visits.

Methodological limitations of the study included a low baseline number of ED visits for ACSCs. Thus, we were not able to assess the impact of the intervention in the population where we anticipated the greatest benefit. In addition, the study was powered for the number of PCPs who participated and not by number of contacts made to SCOPE, which is the unit with the potential to impact the primary outcome of ED visits. Thus the study was not adequately powered for the number of contacts. In addition, we matched for physician characteristics but not patient characteristics. This is a potential limitation if one group of physicians has a sicker patient population, with greater multimorbidity or patients of lower socioeconomic status, which would be more likely to visit the ED. In this study, it would be unlikely that this had a significant impact on the outcome given the overall low number of contacts, as a contact would be needed to prevent an ED visit.

SCOPE is a feasible model of care that was successfully instituted in our local setting with support from local hospitals and has the potential to be scaled to new communities. New services can be added based on unique needs identified by a community. For example in our setting radiology, mental health and a general neurology-headache service have been added based on local need. Patient education at the practice level about their PCP’s access to enhanced resources may also improve effectiveness. If patients perceive that they have expedited access to resources through their PCP, they may be less likely to visit the ED. Incentives directed at PCPs for providing more comprehensive care to patients would also improve sustainability.

## Conclusions

This intervention to support PCPs through timely access to specialist and community resources did not reduce the frequency of ED visits, but solo PCPs felt better supported in the care of their patients. ED use is a complex and multifactorial problem. To improve the likelihood of success of this intervention, the uptake by PCPs would need to increase and become integrated into their way of practicing. This may be more likely in a group of PCPs who are earlier in their career and thus more likely to incorporate it in their daily practice, or in a setting where there are financial incentives to avoid ED visits and inpatient admissions like Accountable Care Organizations.

## Supporting information

S1 Appendix Qualitative interview guide. (DOCX)

S1 Table Common reasons for contacting SCOPE. (DOCX)

S1 Fig Monthly calls to each element of the SCOPE intervention. (TIF)

S2 Fig Monthly patient results viewed in PRO. (TIF)

S3 Fig Outcomes of contacts to SCOPE (n = 1,588). (TIF)

S4 Fig Specialist referrals (n = 282). * Other includes: hematology, ENT, pain management, allergy, plastic surgery, pediatrics, nephrology, ophthalmology and respirology.
(TIF)
