# Supplementary material for: A multifaceted primary care practice-based intervention to reduce ED visits and hospitalization for complex medical patients: A mixed methods study
Source: PLoS One. 2019 Jan 2;14(1):e0209241. doi: 10.1371/journal.pone.0209241 (PMC6314574; doi:10.1371/journal.pone.0209241)
Supplement: S1 Appendix — (DOCX) [file pone.0209241.s001.docx]

**QUALITATIVE INTERVIEW GUIDE**

**Adoption/Initial Use of SCOPE Services**

1. Why did you decide to participate in the SCOPE project?

2. Was there anything that influenced your decision to participate?

a. Recruitment techniques?

b. Involvement of colleagues?

c. Resources offered in association with participation?

d. Opportunities offered in association with participation?

3. Did you perceive any ‘downside’ associated with participating in SCOPE/trying the services?

a. Time?

b. Resources?

4. What was it like using the SCOPE services the first time?

a. Was there anything that encouraged you to try the services?

b. Was there something good or bad about what you had to do to use the SCOPE services for the first time?

5. If you feel comfortable, using a sample anonymized case from your practice, could you walk me through a one of the first cases that you thought it would be beneficial to interact with SCOPE about?

**Ongoing Use of SCOPE Services - Facilitators/Barriers**

6. What types of patients and conditions do you think the SCOPE services are most helpful for?

7. Has participating in SCOPE changed your ability to care for your complex medical patients?

a. If so, how?

b. Are there other ways in which SCOPE has affected your ability to practice? Either positively or negatively?

8. To what extent do the services currently meet your needs?

a. What, if anything, about the services make them useful to you?

b. Could they be modified at all to better meet your needs? Your patients’ needs?

9. In your opinion, how could SCOPE be improved to reduce ED use by patients with complex conditions?

a. Where do you believe the project should focus its efforts in order to provide the most benefit to community PCPs regarding the management of patients with complex conditions?

10. Do you continue to face challenges in caring for your complex patients?

a. If yes, could you tell me about the challenges that you continue to experience?

11. To what extent do you see yourself continuing to use or increasing your use of SCOPE services?

a. Why is this the case?

b. Is there anything good or bad about what you have to do to use the SCOPE services?

12. Do you think that you could using the SCOPE more than you currently are?

13. Have your impressions about the SCOPE project/services changed over time?

a. If yes, how?

14. How do you envision the SCOPE services after the completion of the project?

a. Do you anticipate that they will change? If yes, how?

15. In general, what do you believe are some of the considerations that PCPs think about both when adopting new services and continuing to engage in quality improvement initiatives like SCOPE?

**Recommendations / Wrap Up**

16. In your opinion, do the services offered through the SCOPE project have promise?

a. Could you elaborate on why you believe that?

17. What are some ways that you think we could best measure the impact that SCOPE has had, if any, on your practice and/or patients?

18. Do you have any general recommendations regarding the implementation of quality improvement initiatives like SCOPE?

19. Do you have any additional comments or questions?

**Thank you very much for participating in this interview. Both your time and insights are much appreciated.**

Actual Stop Time of Interview: ___________________________________

**NOTES:**

Distractions/Interruptions:

Visual Cues:

Other Notes:
