# Supplementary material for: A multifaceted primary care practice-based intervention to reduce ED visits and hospitalization for complex medical patients: A mixed methods study
Source: PLoS One. 2019 Jan 2;14(1):e0209241. doi: 10.1371/journal.pone.0209241 (PMC6314574; doi:10.1371/journal.pone.0209241)
Supplement: S1 Table — (DOCX) [file pone.0209241.s002.docx]

**S1 Table:** Common reasons for contacting SCOPE

|  | **N (%)** |
| --- | --- |
| Request for community/homecare assessment | 150 (14%) |
| Diabetes | 130 (12%) |
| Mental Health conditions | 126 (12%) |
| Abnormal test results | 90 (8%) |
| General request for information | 75 (7%) |
| Cardiac conditions | 69 (6%) |
| Addictions/substance abuse | 63 (6%) |
| Cellulitis | 52 (5%) |
| Congestive Heart Failure | 43 (4%) |
| Infection/Fever | 42 (4%) |
| Neurological conditions | 39 (4%) |
| Dementia | 38 (4%) |
| Musculoskeletal conditions | 37 (4%) |
| Respiratory conditions | 36 (3%) |
| Deep Venous Thrombosis | 33 (3%) |
